# Genetic enhancement: an avenue to combat aging-related diseases

**DOI:** 10.1093/lifemedi/lnac054

**Published:** 2022-11-22

**Authors:** Yusheng Cai, Zhejun Ji, Si Wang, Weiqi Zhang, Jing Qu, Juan Carlos Izpisúa- Belmonte, Guang-Hui Liu

**Affiliations:** State Key Laboratory of Membrane Biology, Institute of Zoology, Chinese Academy of Sciences, Beijing 100101, China; Institute for Stem Cell and Regeneration, Chinese Academy of Sciences, Beijing 100101, China; Beijing Institute for Stem Cell and Regenerative Medicine, Beijing 100101, China; Institute for Stem Cell and Regeneration, Chinese Academy of Sciences, Beijing 100101, China; Beijing Institute for Stem Cell and Regenerative Medicine, Beijing 100101, China; State Key Laboratory of Stem Cell and Reproductive Biology, Institute of Zoology, Chinese Academy of Sciences, Beijing 100101, China; Advanced Innovation Center for Human Brain Protection, National Clinical Research Center for Geriatric Disorders, Xuanwu Hospital Capital Medical University, Beijing 100053, China; Aging Translational Medicine Center, International Center for Aging and Cancer, Beijing Municipal Geriatric Medical Research Center, Xuanwu Hospital, Capital Medical University, Beijing 100053, China; CAS Key Laboratory of Genomic and Precision Medicine, Beijing Institute of Genomics, Chinese Academy of Sciences, Beijing 100101, China; China National Center for Bioinformation, Beijing 100101, China; Institute for Stem Cell and Regeneration, Chinese Academy of Sciences, Beijing 100101, China; Beijing Institute for Stem Cell and Regenerative Medicine, Beijing 100101, China; State Key Laboratory of Stem Cell and Reproductive Biology, Institute of Zoology, Chinese Academy of Sciences, Beijing 100101, China; Altos Labs, San Diego, CA 92121, USA; State Key Laboratory of Membrane Biology, Institute of Zoology, Chinese Academy of Sciences, Beijing 100101, China; Institute for Stem Cell and Regeneration, Chinese Academy of Sciences, Beijing 100101, China; Beijing Institute for Stem Cell and Regenerative Medicine, Beijing 100101, China; Advanced Innovation Center for Human Brain Protection, National Clinical Research Center for Geriatric Disorders, Xuanwu Hospital Capital Medical University, Beijing 100053, China

**Keywords:** genetic enhancement, gene editing, stem cell, cell therapy, aging

## Abstract

Aging is a major risk factor for multiple diseases, including cardiovascular diseases, neurodegenerative disorders, osteoarthritis, and cancer. It is accompanied by the dysregulation of stem cells and other differentiated cells, and the impairment of their microenvironment. Cell therapies to replenish the abovementioned cells provide a promising approach to restore tissue homeostasis and alleviate aging and aging-related chronic diseases. Importantly, by leveraging gene editing technologies, genetic enhancement, an enhanced strategy for cell therapy, can be developed to improve the safety and efficacy of transplanted therapeutic cells. In this review, we provide an overview and discussion of the current progress in the genetic enhancement field, including genetic modifications of mesenchymal stem cells, neural stem cells, hematopoietic stem cells, vascular cells, and T cells to target aging and aging-associated diseases. We also outline questions regarding safety and current limitations that need to be addressed for the continued development of genetic enhancement strategies for cell therapy to enable its further applications in clinical trials to combat aging-related diseases.

## Introduction

Aging is a process of gradual functional decline, such as loss of mental or physical abilities, in individuals as they age [1, 2]. During aging, the incidence of senile diseases, including neurodegenerative diseases, cardiovascular diseases, osteoarthritis, and cancer, has increased significantly [2–5]. Globally, the number of old people is rising at an unprecedented rate, bringing great challenges to medical and social communities [6–8]. These deleterious aging outcomes for individuals and societies have led researchers to investigate interventions via a variety of approaches [7, 9–11].

With the advancement of gene editing technologies, scientists can directly edit mutated genes *in vitro* and *in vivo* [12–15]. Thereafter, gene therapy has been applied in pre-clinical trials to treat many diseases, including age-associated diseases [16–18]. However, aging is accompanied by a comprehensive decline in the functions of multiple organs, which can hardly be attributed to and therefore not fixed by a single gene. Moreover, off-target or other side effects of gene therapy may cause unpredictable and devastating outcomes, especially for the older adults, such as lethality or mental disabilities, e.g., the Gelsinger case [19]. Instead of fixing a specific gene, another way to combat aging and its related diseases is cell therapy, which is a cell transplantation-based strategy [20]. Functional decline or loss of stem cells is a hallmark of aging, and supplementation or rejuvenation of these cells is a potential treatment to reverse aging [21]. For example, mesenchymal stem cells (MSCs), with the differentiation potentials into various mesoderm-derived tissue cells and ability to secret cytokines to modulate the surrounding niche, can be transplanted to the injured site and promote wound healing [22–24]. Therefore, MSCs are widely used in regenerative medicine [25–28]. In addition, induced pluripotent stem cells (iPSCs), which are a reprogrammed product of somatic cells, have been widely used in disease modeling, drug screening, and regeneration therapies [29–31]. Because iPSCs can be obtained directly from cells of patients, their autologous transplantation greatly reduces the possibility of immune rejection and avoids the ethical challenges of using embryonic stem cells (ESCs) [32, 33]. In recent years, combined with gene editing technology, iPSCs have been widely used to establish diverse human disease models, including genetic disorders, infectious diseases, and cancer [34–37].

However, due to the harsh microenvironment at the transplant sites, especially in aging and/or diseased microenvironment, cell therapy is confronted with a lot of problems, such as a low survival rate and undermined function after transplantation [38]. For example, studies have shown that MSCs have a survival rate as low as 1% just one day after transplantation [23]. Also, the application of chimeric antigen receptor modified T (CAR-T) cells and other immunotherapy methods in the clinical treatment of cancer have been unsatisfactory due to low survival, non-specific targeting, and compromised activity of reprogrammed cells after transplantation [39, 40]. Furthermore, it has been reported that, during the treatment of aging-associated neurodegeneration via neural stem cell transplantation, a formation of brain tumor or tremendous cell death occurs [41]. Therefore, it is imperative to develop new strategies to improve the safety and efficacy of cell therapy in the aging field.

**Figure 1.**
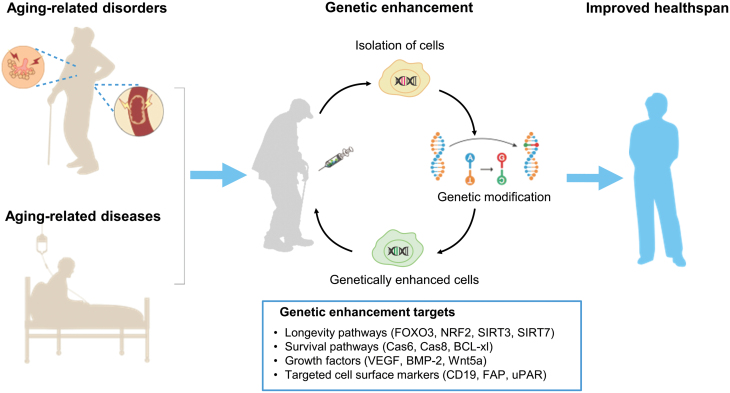
Schematic of genetic enhancement to treat aging-related diseases. After the isolation of selected cells from patients, a specific gene was edited to enhance the viability and functionality of cells. Subsequently, these genetically enhanced cells are transplanted back into the patient. Targeting genes include genes related to longevity pathways, cell survival pathways, growth factors, and cell surface markers.

In order to solve this problem, researchers showed that autologous or heterologous transplanted cells could be genetically enhanced by gene editing, boosting their viability or functionality [42–45]. The treatment with optimized cells is referred to as “genetic enhancement,” which can improve the survival and function of transplanted cells with minimal side effects. Specifically, genetic enhancement modifies the genome of “normal” cells and enhances their function to resist harsh microenvironments in aged or diseased individuals (Fig. 1) [44, 45]. In this review, we summarize the existing strategies and examples of genetic enhancement, focusing on their molecular mechanisms and potential applications for treating aging-related diseases. We further discuss the ethics and future direction of genetic enhancement strategies.

## Genetically enhanced MSCs to treat aging-related diseases

MSCs, also known as mesenchymal stromal cells or mesenchymal progenitor cells, are a type of stromal stem cell with the capacity to self-renew and differentiate into multiple lineages, including osteoblasts, adipocytes, and chondroblasts [46, 47]. MSCs contribute directly to the homeostatic maintenance of the organs in which they reside, and their exhaustion or dysfunction is considered one of the driving forces for deleterious changes in organs during aging [26]. These multipotent stem cells are an attractive option for cell therapy due to their broad sources, and paracrine and autocrine capabilities for immunomodulation and tissue repair [48]. MSCs can be easily isolated from bone marrow (BM), adipose tissue, umbilical cord matrix, dental pulp, and others, or derived from ESCs or iPSCs. Additionally, implanted MSCs secrete growth factors, cytokines, and other trophic factors, which exhibit pro-angiogenic, anti-inflammatory, and anti-fibrotic activities, as well as immunomodulatory effects. MSC therapy provides a promising avenue to increase regenerative capacities and treat different disorders such as osteoarthritis, acute lung injury, cardiovascular diseases, and various central nervous system (CNS) diseases and injuries.

However, the efficacy of MSC therapy is limited by their low survival rate, and their muted *in vivo* functionality and capacity of homing to injured sites [49]. In an attempt to overcome these limitations, genetic enhancement strategies were applied. To improve the survival of MSCs, inhibition of apoptotic pathways and enhancement of pro-survival pathways have been developed. For instance, small hairpin RNA (shRNA) delivered by adenovirus in BM-derived MSCs has been used to reduce the expression of *CASP8* and upregulate *HGF, IGF-1*, and *Bcl-2*, thereby inhibiting MSC apoptosis [38]. Importantly, transplantation of *CASP8* shRNA-modified human MSCs (hMSCs) attenuated cardiac fibrosis and improved heart function after myocardial infarction (MI). Specifically, the ejection fraction was recovered from 47.2% (MSC-*Vector*) to 74.6% (MSC-*CASP8* shRNA) [38]. On the other hand, Akt (Protein kinase B, PKB), as a serine/threonine kinase, plays an important role in cell survival [50]. BM-derived MSCs overexpressing *Akt* (*Akt*-MSCs) antagonized hypoxia-induced apoptosis and therefore were endowed with improved capacity of myocardial regeneration through paracrine mechanisms [51, 52]. Notably, transplantation of *Akt-*MSCs derived from BM restored four-fold greater myocardial volume than that of MSCs carrying vector [52]. Additionally, growth arrest-specific (Gas6), a secreted vitamin K-dependent protein, is involved in the regulation of cell proliferation, mitogenesis, and cell survival [53]. MSCs derived from BM engineered to overexpress *Gas6*, displayed alleviated apoptosis, improved survival and extended retention of MSCs *in vivo* after transplantation [54]. The survival rate of transplanted cells and left ventricular function were improved remarkably in the *Gas6*-MSC treated rats than that of control MSCs after MI [54].

In addition to the increased survival rate, the function of MSCs, including differentiation efficiency and paracrine effect, can also be improved by genetic enhancement to alleviate age-associated tissue degeneration. For example, bone morphogenetic protein 2 (BMP-2) is an important factor that promotes the differentiation of MSCs into osteoprogenitor cells and bone formation [55, 56]. Genetically modified BM MSCs overexpressing *BMP-2* facilitated the induction of osteoblasts and enhanced bone repair and bone regeneration *in vivo* [57]. Meanwhile, genetic enhancement can also improve the paracrine signaling of MSCs, promoting the angiogenesis and neurogenesis. For angiogenesis, both vascular endothelial growth factor (VEGF) and angiogenin (ANG) stimulate angiogenesis [58, 59]. Transplantation of *VEGF*-overexpressing BM MSCs in the hippocampus improved neovascularization and decreased senile plaques in a murine model of Alzheimer’s disease (AD), demonstrating a therapeutic effect of repairing vascular damage in neurodegenerative diseases [60]. BM MSCs with a hypoxia-inducible production of VEGF, named as hypoxia-inducible VEGF expression MSCs (HI-VEGF-MSCs), enhanced the retention of transplanted cells at the infarcted site and improved the repair of MI [61]. For neurogenesis, neurotrophins, glial cell line-derived neurotrophic factor (GDNF), and nerve growth factor are neurotrophic factors that are essential for the repair of injured neurons. The injection of *GDNF*-modified MSCs derived from rat BM can significantly increase the mean dopamine level in the striatum of lesioned rats and attenuate symptoms of Parkinson’s disease (PD) rats [62].

**Figure 2.**
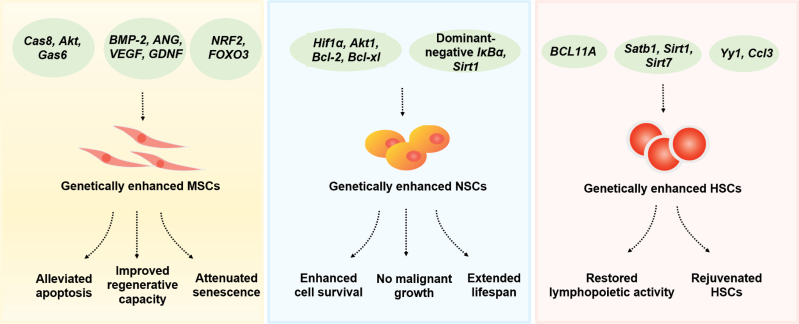
Genetic enhancement of MSCs, NSCs, and HSCs. Stem cells can be enhanced in many ways, including engineered overexpression, suppression, or modification of individual genes and pathways. Multiple types of genetically enhanced stem cells with better survival rate and functionality showed intervention potentials for aging or aging-related diseases. MSCs, NSCs, and HSCs refer to mesenchymal stem cells, neural stem cells, and hematopoietic stem cells, respectively.

Instead of upregulating or downregulating a specific gene, genetic enhancement can also be achieved by editing specific nucleotides of the target gene. Previously, researchers found NRF2 as an important transcription factor in counteracting oxidative stress and cytoprotection [63]. In addition, through the alteration of one nucleotide of *NRF2* by gene editing, the change of glutamate 82 to glycine (E82G) in the NRF2 protein improved the stability and transcription activity of NRF2 [45]. *NRF2*-enhanced hMSCs derived from hESCs exhibited increased self-renewal ability, improved stress resistance, and retarded cellular senescence [45]. Moreover, *NRF2* enhancement endowed MSCs with increased survival and vascular regeneration capacity at ligation site of mice with hindlimb ischemia. More importantly, even in the presence of a panel of strong oncogenic stimuli, *NRF2*-enhanced MSCs did not form any tumor even 10 weeks after the implantation, while wild type transformed MSCs generated palpable tumors within 6 weeks, suggesting the lowered oncogenic transformation of *NRF2*-enhanced hMSCs [45]. This work provides a proof-of-concept to generate superior and safer stem cells for future genetic enhancement cell therapy. Later, leveraging single-cell sequencing techniques, researchers captured the aging landscapes of the aorta and coronary arteries in non-human primates and found that FOXO3A, a longevity-associated transcription factor and a geroprotector for vascular cell aging, was downregulated in aged vascular cells [64]. Based on this clue, genetically enhanced human MSCs by recoding two nucleotides of *FOXO3* to activate its activity constitutively were developed. This genetic enhancement strategy reinforced the MSCs with more robust regenerative capacities and reduced the risk of tumorigenesis. Additionally, these cells exhibited increased resistance to multiple stresses and cellular senescence. Transplantation of *FOXO3*-enhanced hMSCs improved revascularization and the recovery of blood flow in a hindlimb ischemia mouse model [44]. Moreover, *FOXO3*-enhanced human MSCs could effectively promote cardiac repair after mouse MI [65]. Taken together, these studies demonstrate the superiority and safety of MSC genetic enhancement strategies, opening a new avenue for the stem cell treatment of aging-related diseases (Fig. 2).

## Genetically enhanced NSCs to attenuate neurodegeneration

Neurodegeneration during aging leads to a progressive functional decline of older adults’ cognitive, social, and physical abilities, which significantly impacts their life quality [66]. Moreover, the associated neurodegenerative diseases, like AD, PD, and amyotrophic lateral sclerosis, cause severe disability and, ultimately, millions of deaths worldwide [67]. However, there is no effective therapy to stop or reverse the progression of aging-induced degeneration in the neuronal system by classical drug-based treatments. Therefore, genetic enhancement strategies that can reactivate or enhance aged neural cell function could be a promising solution.

NSC transplantation is a widely used approach to antagonize neurodegenerative disorders during aging [68–70]. Several studies have revealed that transplanted human NSCs (hNSCs) can give rise to new neurons and glial cells to repair the damaged region in disease models [68, 70]. For example, in the AD mouse models, transplanted hNSCs extensively migrated and engrafted, and some of them differentiated into new neuronal and glial cells. After hNSC transplantation, the AD model mice displayed improved spatial memory and decreased tau phosphorylation compared to the control group, indicating the potential beneficial effects of hNSC therapy in AD treatment [68, 70].

However, some side effects associated with NSC transplantation have been reported, such as a large amount of cell death or brain tumor formation after NSC transplantation [71, 72]. To solve these problems, researchers employed genetic enhancement strategies. For example, they separately expressed pro-survival factors, including HIF1A, AKT1, BCL2, or BCL2L1, in transplanted NSCs and enhanced their survival rates without growth of malignant tumors [73]. Additionally, other reports showed that overexpressing *Wnt5a* and miRNA200b-3p in transplanted NSCs promoted the differentiation capacity during the treatment of spinal cord injury in mice [74]. Taken together, these genetically enhanced NSCs could act as more efficient and safer tools for preventing and curing neurodegeneration-associated diseases.

In addition to aging-associated diseases, recent studies have shown that genetic enhancement in specific brain regions could delay the aging process of the whole body. Hypothalamus is a small but essential region of the brain located above the brainstem, regulating many essential physiological functions, such as reproduction, growth, metabolism, and homeostasis [75–77]. Researchers reported an age-related activation of nuclear factor κB (NF-κB) and its upstream IκB kinase-β (IKK-β) in the hypothalamus [77]. Knocking out *IKK-β* resulted in a lifespan extension and improved geroprotective phenotypes in mice [77]. Thereafter, it has been revealed an age-related loss of hypothalamic neural stem/progenitor cells (htNSCs). Additionally, removing htNSCs in mouse models leads to shortened lifespan and aging-like physiological decline, suggesting htNSCs as a potential target for genetic enhancement treatment [78]. To address this, mid-aged mice implanted with htNSCs stably expressing dominant-negative IκBα (IκBα-htNSC), continuously inhibiting the NF-κB pathway, exhibited aging retardation and lifespan extension. Other findings also showed that modifications of certain genes’ expression in the brain, such as *Sirt1*, could extend lifespan and promote overall fitness in mice [76, 79]. However, the detailed mechanisms behind this phenomenon still need to be investigated. In general, NSCs could serve as a potential target in the neuronal system for genetic enhancement treatment to combat aging and neurodegeneration.

## Genetically enhanced vascular cells to alleviate aging defects

The cardiovascular system circulates blood, oxygen, and nutrients around the body, and consists of the heart and a complex network of blood vessels [80]. Age-associated dysfunction in the cardiovascular system gradually affects the fitness of all organisms and ultimately induces heart failure [81]. Therefore, it is essential to maintain the health of the cardiovascular system. So far, several genetic enhancement strategies have been developed to target different cells within the cardiovascular system, including vascular cells [82–85].

Human blood vessels are mainly composed of three tissue layers, namely the inner layer, tunica intima, which comprises vascular endothelial cells (VECs); the middle layer, tunica media, which consists of vascular smooth muscle cells (VSMCs) intermingled with elastic fibers; and the external layer, tunica adventitia, which contains an extracellular matrix (collagen and elastin) and loose fibrous connective tissue [86]. Importantly, vascular aging is often associated with dysfunction of endothelial cells (ECs), impaired cellular structure of VSMCs, and disorganized extracellular matrix degradation [86, 87]. In order to ameliorate the functional decline of these cells, researchers have genetically modified vascular cells and investigated their effects *in vivo* and *in vitro* [44, 83]. FOXO3 is the main downstream effector of the PI3K/Akt pathway and contributes to human longevity regulation [88]. Continuous activation of FOXO3 in human VECs and human VSMCs endows them with anti-oxidant activity and senescence-resistant capacity [44]. In addition, FOXO3-engineered human VECs and human VSMCs exhibited improved vascular regeneration potential compared to wild-type cells when used to treat limb ischemia in murine models [44, 89]. Therefore, the transplantation of genetically enhanced vascular cells presents a promising path to alleviate aging and aging-associated cardiovascular diseases.

## Genetically engineered HSCs to promote their rejuvenation

Several studies have reported age-related exhaustion, impaired self-renewal ability, and declined homing efficiency of hematopoietic stem cells (HSCs), which induce immunodeficiencies, increases anemia along with the reduced HSC reconstitution capacity [90–92]. This deterioration of the hematopoietic system ultimately leads to chronic inflammation, vascular diseases, and tumorigenesis in the cardiovascular system of older adults [93, 94]. Therefore, researchers proposed to rejuvenate the HSC pool by transplantation of young HSC resources [95, 96]. However, there are also limitations, such as restricted availability and compatibility of young HSC resources [97]. To address this, researchers proposed heterochronous autologous hematopoietic stem cell transplantation (haHSCT), in which HSCs were collected during the healthy period of life and kept in liquid nitrogen for several years to potentially treat age-related diseases later in the same person [98]. This strategy will not cause immune rejection since these young HSCs are derived from the same individual. Furthermore, young populations of HSCs can partially replace the older population *in vivo* and thus are beneficial to maintain healthy aging [99].

However, haHSCT is not optimal for elderly patients or patients with inherited diseases, and thus genetic modifications of autologous HSCs are attractive. For example, patients with inherited diseases, such as β-hemoglobin (*HBB*) disorders, which are caused by a single gene mutation, encounter severe morbidity and mortality [100]. In order to generate HSCs with persistent hemoglobin induction, researchers constructed *BCL11A* enhancer-edited hematopoietic stem and progenitor cells (HSPCs) collected from patients with *HBB* disorders. They transplanted these genetically modified stem cells into rhesus monkeys and found that they produced persistent hemoglobin levels in peripheral blood and BM, suggesting a potential genetic treatment for humans with inherited disorders [101].

Similarly, the genetic enhancement could be used to rejuvenate the aged HSC pool to antagonize aging. Several reports showed an age-associated down-regulation of specific genes in HSCs, like *Satb1*, *Sirt3*, and *Sirt7*, whereas overexpression of these genes can rejuvenate HSCs [102–104]. Among them, *SATB1* is a chromatin regulator that functions during lymphoid lineage specification [105]. Overexpression of *Satb1* in HSPCs collected from old mice can partially restore their lymphopoietic activity [104]. In addition, SIRT3 is an NAD^+^-dependent histone deacetylase that reduces reactive oxygen species in aged HSCs, and overexpression of *Sirt3* can rejuvenate aged HSCs [102]. Similarly, overexpression of *Sirt7*, another gene coding a histone deacetylase of the sirtuin family, was reported to improve the regenerative function of senescent HSCs [103]. Moreover, overexpression of the identified rejuvenating factors, including *Ccl3* or *Yy1*, could attenuate the age-associated lymphopoiesis decline [106, 107]. Taken together, genetically enhanced HSCs are promising resources for transplantation to promote healthy aging and treat aging-associated disorders.

## Genetically modified/engineered T cells in disease intervention

Immune cells regulate the immune response, including innate and adaptive immune responses, to defend against a wide variety of pathogens and toxins [108]. The immune system plays an essential role in maintaining homeostasis, and its dysregulation is tightly associated with cancer development and aging. Combining synthetic biology techniques with gene editing, immune cell therapies, including T cells, natural killer cells, and macrophages (monocytes) show great promise for treating cancer and other age-related diseases.

### Targeting cancer cells with CAR T cells

T cells are essential in adaptive immunity and express highly variable surface markers to recognize foreign particles (antigens). Genetically engineered T cells were developed to selectively target and treat cancer [39, 109–111]. CARs are recombinant receptors that allow T cells to recognize antigens on targeted tumor cells [112–114]. For example, CD19, universally and ubiquitously expressed on B-cell surfaces and involved in nearly all B-cell malignancies, is an attractive target for CAR-T cells for the treatment of B-cell malignancies. Clinical trials of CD19-targeting CAR T cells showed promising results in chronic lymphocytic leukemia and acute lymphoblastic leukemia and acquired U.S. Food and Drug Administration approval [115]. In addition, other antigens, including B-cell maturation antigen (BCMA), CD20 for B cells and CD5 as well as CD7 for T cells, have been developed to target multiple myeloma [116].

To further enhance the anticancer effects of T cells, antigen-redirected T cells were modified to ameliorate the exhaustion of T cells, overcome immunosuppression and modulate the immune cell responses and microenvironment [116, 117]. To reduce the exhaustion of T cells, the anti-apoptotic genes, *BCL-XL* and *BCL-2*, were selected to inhibit cell apoptosis and improve the survival rate of T cells [118]. To overcome the immunosuppressive environment of different cancers, the Synthetic Notch (SynNotch) receptor was expressed in T cells to modulate the immune cell responses and remodel the microenvironment of different diseases [119]. Additionally, CAR-T cells have been engineered to express human anti-programmed death ligand 1 (PD-L1) antibodies or PD-1-blocking single-chain variable fragments (scFv), improving the efficacy of the CAR T cells [117, 120]. Moreover, Interleukin-12 overexpression in T cells has also been used to stimulate the proliferation of T cells, modify regulatory cells like myeloid cells, and change the microenvironment to increase sensitivity to the immune response [121].

### Targeting fibrosis with engineered T cells

Fibrosis is a degenerative phenotype of tissue aging caused by excessive extracellular matrix deposition, which disrupts normal tissue architecture and impairs organ function [122, 123]. Cardiac fibroblasts, an important component of the heart, can transform into activated pathological fibroblasts upon injury and secrete excessive extracellular matrix leading to tissue stiffness, fibrosis and ultimately heart failure. The fibroblast activation protein (FAP) is a cell-surface glycoprotein that is robustly upregulated in cardiac fibroblasts after different injuries or in patients with cardiac diseases [124, 125]. Indeed, FAP-specific CD8^+^ T cells can target cardiac fibrosis and improve cardiac functions of heart-injured mice [124]. Collectively, engineered T cell therapy not only shows great promise in oncology but also provides a novel and promising strategy for fibrosis interventions.

### Eliminating senescent cells with engineered T cells

The aberrant accumulation of senescent cells contributes to chronic inflammation, tissue dysfunction, age-associated chronic diseases, and tumorigenesis in aged organisms [126, 127]. Senolytic drugs have been developed to specifically target senescent cells and ameliorate the symptoms of multiple pathologies and restore physical functions of aged mice [128]. Recently, the surface protein urokinase-type plasminogen activator receptor (uPAR) was identified as a universal senescent marker and explored as a CAR T cell target to delete senescent cells [129]. uPAR-specific CAR T cells effectively ablated senescent cells both *in vitro* and *in vivo*, and reduced liver fibrosis induced by carbon tetrachloride (CCl_4_) or nonalcoholic steatohepatitis (NASH) resulting in an improvement of liver functions. These CAR T cells targeting uPAR also improved the survival rate of mice with lung adenocarcinoma combined with senescence-inducing drugs [129]. Thus, engineered CAR T cells targeting senescent cell surface markers have potential value for the treatment of senescence-associated diseases.

## Current limitations and ethical issues

Although genetic enhancement could serve as a potent strategy to treat multiple aging-related diseases, there are also several technical limitations [130]. First, considering the off-target effects, which may cause point mutations, insertions, translocations in genome, and so on, gene editing tools should be modified and engineered to obtain higher efficiency and specificity to avoid genotoxic effects [131, 132]. Second, since the viral delivery tools might have safety concerns, researcher proposed to use non-viral vectors, such as lipid-based nanoparticles [133, 134]. However, the delivery efficiency of non-viral vectors is cell-type dependent [135, 136]. Therefore, safer and more efficient delivery tools should be developed in the future. Third, to maintain the integrity of the genome, the genetic modification, such as changes in the number of nucleotides, should be as few as possible. Last but not least, ethical issues should be sincerely addressed in the field. All genetic enhancement technologies discussed in this review do not involve any germ-line editing or adults without consent. Collectively, before applying to clinical practices, genetically enhanced cell therapy should be strictly quality-controlled, technically monitored, and ethically discussed case by case [17].

## Conclusions and perspectives

**Figure 3.**
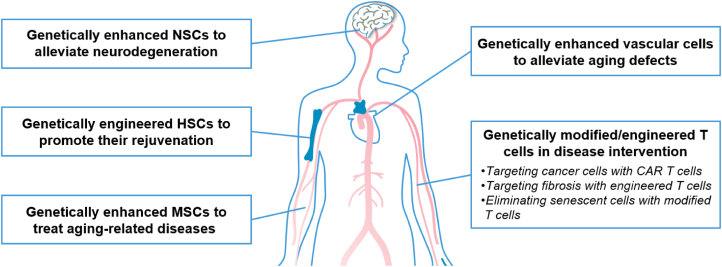
Schematic of the potential applications of genetic enhancement in various types of cells. Genetically enhanced cells can be transplanted to different locations within diverse human systems. Prior studies have tested several systems, including the nervous system, immune system, and cardiovascular system, as well as other systems. MSCs, NSCs, and HSCs refer to mesenchymal stem cells, neural stem cells, and hematopoietic stem cells, respectively.

Genetic enhancement strategies endow stem cells and differentiated cells with improved safety and efficacy [44]. Through the modification of target genes, their regenerative capacity can be improved, which enhances their therapeutic effect (Table 1). In this review, we have outlined current progress in genetic enhancement of MSCs, NSCs, vascular cells, HSCs, and T cells (Fig. 3). We also summarized the outcome of genetic enhancement via different strategies, including improved cell survival, regeneration, differentiation potentials, and safety. Furthermore, we discussed the limitation and safety of genetic enhancement, which should be carefully monitored before applying to clinical practices. With the advancement of understanding longevity pathway, healthy aging regulation, stress resistance, and anticancer mechanisms, researchers will develop more enhancement strategies to revolutionize cell therapy fields [137]. In the future, genetically enhanced cells will potentially serve as an advanced cell resource in the cell therapy field, providing powerful, standardized and commercialized cell therapy products to treat multiple age-associated disorders and diseases.

**Table 1.** Summary of genetically enhanced strategies in different cell types with different targets to treat diseases

Cell type	Target gene	Treatment	Gene vector	Enhanced function	Target disease	Refs
**MSCs**	*CASP8*	Knockdown by shRNA	Adenovirus	Inhibit the MSC apoptosis	Attenuate cardiac fibrosis and improve heart function after myocardium infarction	Liang et al.
	*Akt*	Overexpression	Retrovirus	Inhibite hypoxia-induced apoptosis	Improve myocardial regeneration through the paracrine mechanisms	Gnecchi et al.
	*Gas6*	Overexpression	Adenovirus	Alleviate MSC apoptosis	Improve cell survival, enhance cardiac function in a rat model of MI	Shan et al.
	*BMP-2*	Overexpression	Gene-transfection by APA microcapsules	Induce osteoblasts	Facilitate bone repair and bone regeneration	Ding et al.
	*VEGF*	Conditional overexpression	Adenovirus	Improve the left ventricle ejection fraction and fractional shortening in a rat MI model	Improve the repair of myocardial infarction	Kim et al.
	*VEGF*	Overexpression	Sleeping beauty transposon vector	Improve the neovascularization and diminished senile plaques in hippocampal specific layers	Repair the vascular damage in the neurodegeneration diseases, including AD	Garcia et al.
	*Angiogenin*	Overexpression	Adenovirus	Improve their viability	Preserve heart function by vasculogenesis	Liu et al.
	*Neurotrophins, GDNF or NGF*	Overexpression	Lentivirus	Enhance the neural regeneration	Treat multiple degenerative diseases, including Parkinson’s disease	Shi et al.; Rooney et al.
	*NRF2*	Genome editing at target gene nucleotides	Helper-dependent adenoviral vector (HDAdV)	Exhibit increased self-renewal ability, improve stress resistance, and anti-senescence	Increase regenerative capacity after hindlimb ischemia and lower oncogenic transformation	Yang et al.
	*FOXO3*	Genome editing at target gene nucleotides	Adenovirus	Prevent cellular senescence and improve resistance to oxidative stress	Promote revascularization after mouse hindlimb ischemic; promote cardiac repair after mouse myocardial infarction	Yan et al.
**NSCs**	*Hif1a, Akt1, Bcl2 or Bcl2l1*	Overexpression	Lentivirus	Improve the survival rate of transplanted NSCs	Prevent stroke-induced behavioral outcome and recover motor activity	Korshunova et al.
	*Wnt5a*	Overexpression	Lentivirus	Improve the differentiation rate of NSCs	Promote NSC differentiation into neurons to promote motor functional and histological recovery after SCI	Schaum et al.
	*miRNA200b-3p*	Overexpression	Lentivirus	Improve the differentiation rate of NSCs	Promote NSC differentiation into neurons to promote motor functional and histological recovery after SCI	Schaum et al.
**VECs**	*FOXO3*	Overexpression	Adenovirus	Prevent cellular senescence and improve resistance to oxidative stress	Promote revascularization after ischemic surgery	Yan et al.
**VSMCs**	*FOXO3*	Overexpression	Adenovirus	Prevent cellular senescence and improve resistance to oxidative stress	Promote revascularization after ischemic surgery	Yan et al.
	*BMP4*	Suppression	BMP4 antibody blocking	Recover the functionality of the vascular barrier *in vitro*	Limit cardiovascular dysfunction in HGPS	Bersini et al.
**HSCs**	*Satb1*	Overexpression	Retrovirus	Enhance B lineage growth from HSCs	Restore lymphoid potential in aged hscs	Satoh et al.
	*Sirt3*	Overexpression	Lentivirus	Improve the regenerative capacity of HSCs	Reverse HSC aging	Brown et al.
	*Sirt7*	Overexpression	Lentivirus	Improve the regenerative capacity of aged HSCs	Reverse HSC aging	Mohrin et al.
**T cells**	*CD19*	Overexpression	Retrovirus	Target B-cell malignancies	Treat chronic lymphocytic leukemia (CLL) and acute lymphoblastic leukemia (ALL)	Rafiq et al.; Maude et al.
	*FAP*	Overexpression	Retrovirus	Target and delete cardiac activated pathological fibroblasts to reduce fibrosis	Target cardiac fibrosis and improve cardiac functions of heart-injured mice	Aghajanian et al.
	*uPAR*	Overexpression	Retrovirus	Target senescent cells	Reduce liver fibrosis induced by CCl4 or NASH resulting in an improvement of liver functions	Amor et al.

Abbreviations: MSCs, mesenchymal stem cells; NSCs, neural stem cells; VECs, vascular endothelial cells; VSMCs, vascular smooth muscle cells; APA microcapsules, alginate-poly-l-lysine microcapsules; MI, myocardium infarction; HI, hindlimb ischemia; FAP, Fibroblast activation protein; uPAR, urokinase-type plasminogen activator receptor; CCl4 subscript, carbon tetrachloride; NASH, nonalcoholic steatohepatitis; AD, Alzheimer’s disease.

## Notes

This work was supported by the National Key Research and Development Program of China (Nos. 2022YFA1103700, 2020YFA0804000), the Strategic Priority Research Program of the Chinese Academy of Sciences (No. XDA16000000), CAS Project for Young Scientists in Basic Research (Nos. YSBR-076, YSBR-012), the National Natural Science Foundation of China (Nos.81921006, 82125011, 92149301, 92168201, 91949209, 92049116, 32121001, 82122024, 82071588), the National Key Research and Development Program of China (Nos. 2018YFC2000100), STI2030-Major Projects-2021ZD0202400, the Program of the Beijing Natural Science Foundation (No.Z190019), and the Tencent Foundation (No. 2021-1045).
